# Construction and validation of a diagnostic model for type 2 diabetes mellitus comorbid with metabolic dysfunction-associated steatotic liver disease based on routine serological indicators: a retrospective case-control study

**DOI:** 10.3389/fendo.2026.1891690

**Published:** 2026-08-21

**Authors:** Lihong Wu, Shuang Wang, Ning Yang, Renjun Li, Shui Jin

**Affiliations:** Chaohu Hospital of Anhui Medical University, Chaohu, China

**Keywords:** type 2 diabetes mellitus, diagnostic model, metabolic dysfunction-associated steatotic liver disease, nomogram, serological indicators

## Abstract

**Objective:**

The prevalence of type 2 diabetes mellitus (T2DM) with concurrent Metabolic dysfunction-Associated Steatotic Liver Disease (MASLD) is high. However, simple, efficient and non-invasive diagnostic tools based on routine serological indicators are lacking. This study aims to construct and validate a simple diagnostic model using routine serological indicators.

**Methods:**

A retrospective case-control study is conducted, enrolling 470 patients with T2DM. They are randomly stratified into a training set and a validation set at a 7:3 ratio. Independent predictive factors are identified using univariate and multivariate logistic regression. Diagnostic performance is assessed via receiver operating characteristic (ROC) curves. A nomogram diagnostic model is constructed and evaluated using ROC curves, calibration curves and decision curve analysis. For external validation, an additional 300 T2DM patients are recruited.

**Results:**

Body mass index (BMI), fasting C-peptide, total cholesterol, triglycerides, high-density lipoprotein cholesterol (HDL), low-density lipoprotein cholesterol (LDL), urea and alanine aminotransferase (ALT) are independent predictors of comorbid T2DM and MASLD. The area under the curve (AUC) of this model in the training set, internal validation set and external validation set is 0.921, 0.913 and 0.830, respectively. The calibration curve closely approximates the ideal diagonal line and decision curve analysis (DCA) demonstrates significant clinical net benefit across a broad range of threshold probabilities.

**Conclusion:**

This model enables high-precision diagnosis of T2DM with MASLD using routinely available serological indicators, avoiding invasive procedures and exhibiting strong potential for clinical translation. Future studies should validate its generalizability in multicenter prospective cohorts to promote its use as a routine screening tool for MASLD in T2DM patients.

## Introduction

Type 2 diabetes mellitus (T2DM) and Metabolic dysfunction-Associated Steatotic Liver Disease (MASLD) are two highly prevalent metabolic diseases worldwide. They frequently coexist, forming a severe “dual metabolic burden” (1). Among T2DM patients, the prevalence of MASLD ranges from 55% to over 70%, a comorbidity rate far exceeding that of the general population (2). This close association is not coincidental. The liver, as a central hub for nutrient and energy metabolism, is central to a mutually reinforcing relationship between lipid deposition and insulin resistance (3). Critically, when T2DM and MASLD coexist, insulin resistance is exacerbated, progression of both micro- and macrovascular diabetic complications accelerates and liver pathology advances from simple steatosis to non-alcoholic steatohepatitis (NASH), fibrosis, cirrhosis and hepatocellular carcinoma. This synergy substantially increases liver-related and cardiovascular mortality (4). Consequently, MASLD has been recognized as an emerging and highly concerning complication of diabetes, imposing a substantial burden on global public health systems and medical economic resources (5). Therefore, early and precise identification of T2DM patients with concurrent MASLD is of critical clinical importance for timely intervention, disease progression arrest and improvement of long-term prognosis.

Currently, diagnostic and assessment methods for MASLD each have notable limitations. Although liver biopsy remains the gold standard for diagnosing NASH and staging fibrosis (5), its invasiveness, sampling errors, procedural risks and high costs preclude its widespread use as a routine screening tool for the large outpatient T2DM population (6). Conventional imaging, such as abdominal ultrasonography, is simple and convenient and serves as the primary clinical first-line method for diagnosing fatty liver. However, it lacks sensitivity for mild hepatic steatosis (<10%) and cannot accurately distinguish simple steatosis from NASH (7). Given these challenges, exploring a non-invasive, convenient, cost-effective and scalable screening strategy is an urgent clinical need. Utilizing serological markers from routine blood tests to construct diagnostic prediction models represents a promising and translatable solution. These specimens are readily available, detection methods are standardized and reproducibility is high, making this approach well-suited for large-scale population screening and primary healthcare settings.

In recent years, the value of conventional serological indicators in assessing metabolic disease risk has been re-evaluated due to their economy, objectivity and reproducibility. Studies have confirmed that abnormalities in liver enzymes, including alanine aminotransferase and aspartate aminotransferase, are independently associated with hepatic steatosis. Meanwhile, lipid disorders such as hypertriglyceridemia and low high-density lipoprotein cholesterol are highly prevalent in MASLD patients (8). Furthermore, novel cell-derived indicators reflecting systemic inflammation, such as the neutrophil-to-lymphocyte ratio and platelet-to-lymphocyte ratio, are emerging as new links between metabolism and immunity. Previous studies have utilized serum bile acid profiles to predict MASLD in T2DM patients, finding that elevations in specific conjugated bile acids can significantly enhance the predictive accuracy of clinical models (9). These findings collectively suggest that integrating a combination of conventional serological markers from multiple systems may be key to addressing current diagnostic challenges.

Although single indicators or multi-dimensional scores have been explored, the systematic integration of routinely available serological tests into a diagnostic model with high discrimination, calibration and clinical utility in the specific context of T2DM and MASLD comorbidity remains an unaddressed research gap. Previous studies have constructed nomograms for predicting metabolic-associated fatty liver disease in newly diagnosed T2DM patients using clinical and laboratory indicators, but they focus on the latest criteria for metabolic-associated fatty liver disease, and some models introduce a large number of variables, which may pose accessibility challenges in busy frontline clinical settings (10). This study aims to maximize diagnostic value from the most routine serological examination items. This approach complements the development of novel, high-cost omics technologies and facilitates promotion in medical institutions at all levels, especially in primary care settings, reflecting the core advantage of clinical translational potential (11).

Based on these considerations, this study adopts a retrospective case-control design to systematically evaluate the comprehensive diagnostic efficacy of conventional serological indicators for T2DM with MASLD. To ensure scientific rigor, subjects are divided into training and validation sets via stratified random sampling to ensure balanced distribution and objectively assess model generalizability. In the variable selection and model construction phase, we plan to jointly apply univariate and multivariate logistic regression analysis and random forest algorithms. This hybrid strategy aims to leverage complementary advantages: logistic regression quantifies the independent contributions of each factor and generates a clinically interpretable risk formula, while diagnostic ROC curves effectively evaluate diagnostic value among variables. This method has demonstrated excellent performance in studies constructing diagnostic models using multiple indicators. The final constructed model is presented as an intuitive nomogram, providing a user-friendly individualized assessment tool for clinical practice.

In summary, the main purpose of this study is to use conventional serological indicators to screen independent predictors of MASLD in T2DM patients and construct a non-invasive, efficient and low-cost diagnostic model. The potential value of this study lies in providing a precise screening strategy that surpasses traditional single indicators or complex scoring systems, thereby promoting early detection and intervention of MASLD and ultimately improving patients’ long-term prognosis.

## Materials and methods

### Study design and subjects

This study employs a retrospective case-control design. Clinical data are collected from 470 hospitalized T2DM patients at the Fourth Affiliated Hospital of Anhui Medical University between January 1, 2023 and December 31, 2024. Patients are stratified based on abdominal ultrasonography findings into the MASLD group (case group) and the non-MASLD group (Non-case group). To further validate the model’s performance, clinical data are additionally collected from 300 hospitalized T2DM patients at the same hospital between January 1, 2021 and December 31, 2022. These patients are similarly stratified based on abdominal ultrasonography findings into the MASLD group (case group) and the non-MASLD group (Non-case group).

### Data collection

Clinical data collect age, sex, height, weight, body mass index (BMI), smoking and hypertension. After an overnight fast of 8–10 hours, venous blood samples are collected from all subjects the following morning. Routine serological indicators included: liver function markers [alanine aminotransferase (ALT), aspartate aminotransferase (AST), gamma-glutamyl transpeptidase (GGT), albumin (ALB) and globulin (Glob)]; lipids [total cholesterol (TC), triglycerides (TG), high-density lipoprotein cholesterol (HDL) and low-density lipoprotein cholesterol (LDL)]; glucose metabolism markers [fasting blood glucose (FBG), HbA1c, fasting C-peptide (C-P) and insulin]; renal function indicators [urea, creatinine (Cr) and uric acid (UA)]; complete blood count indicators [white blood cells (WBC), neutrophil count (Neut), lymphocyte count (Lymph), monocyte count (Mono), hemoglobin (Hb) and platelet count (PLT)] and systemic inflammatory indicators calculated as: NLR = neutrophil-to-lymphocyte ratio; PLR = platelet-to-lymphocyte ratio; MLR = monocyte-to-lymphocyte ratio; SII (systemic immune-inflammation index) = (platelet count * neutrophil count)/lymphocyte count; SIRI (systemic inflammatory response index) = (neutrophil count * monocyte count)/lymphocyte count.

### Inclusion criteria

1. No history of alcohol consumption or weekly alcohol intake <70 g for women and <140 g for men.
2. Abdominal ultrasonography indicating fatty liver.
3. Complete clinical data.

### Exclusion criteria

1. Presence of other liver diseases, including viral hepatitis, alcoholic liver disease, drug-induced liver injury, autoimmune liver disease, cirrhosis or liver cancer.
2. Other metabolic diseases: severe renal insufficiency, thyroid dysfunction, Cushing’s syndrome or other conditions significantly affecting metabolic indicators.
3. Special conditions: pregnancy, lactation, etc.
4. Missing key clinical data or serological indicators precluding complete analysis.
5. Other interfering factors: acute infections, cancers, hematological diseases or recent major surgery or trauma significantly affecting inflammatory and metabolic indicators.

### T2DM diagnostic criteria

The diagnosis of T2DM follows the 2020 Chinese Guidelines for the Prevention and Treatment of Diabetes Mellitus:

A diagnosis is established when classic diabetes symptoms are present alongside any one of the following four criteria:

Random blood glucose ≥ 11.1 mmol/L;

Glycated hemoglobin (HbA1c) ≥ 6.5%;

Fasting blood glucose ≥ 7.0 mmol/L;

2-hour post-load blood glucose ≥ 11.1 mmol/L.

### MASLD diagnostic criteria

Abdominal color Doppler ultrasound serves as the diagnostic modality for hepatic steatosis:

The imaging reveals diffuse enhancement of near-field hepatic echoes, exhibiting greater echogenicity compared to the renal cortex;

There is a progressive attenuation of far-field hepatic echoes observed on the scan;

Intrahepatic biliary structures are poorly visualized on the imaging scan;

A diagnosis of hepatic steatosis is established when at least two of the aforementioned ultrasonographic criteria are met.

### Statistical analysis

After stratification by event proportion, data are randomly divided into a training set and an internal validation set at a 7:3 ratio, using a fixed random seed (123) to ensure reproducibility. All variable selection, model construction and machine learning analyses are performed on the training set, while the internal validation set remain blinded throughout to preliminarily evaluate model generalizability.

First, baseline characteristics are analyzed. For variables following a normal distribution with equal variances, a t-test is used. For variables with a normal distribution but unequal variances, Welch’s t-test is applied. And for non-normally distributed data, the Wilcoxon rank-sum test is used.

Potential predictive factors are initially screened in the training set using univariate logistic regression. Variables with *P* < 0.1 are included in multivariate logistic regression analysis to determine independent predictors. A nomogram prediction model is then constructed based on regression coefficients. Additionally, diagnostic ROC curves are used to evaluate the diagnostic value of each predictive variable.

To assess internal stability and correct for overfitting bias, the model’s discrimination is evaluated using ROC curves within the training set. Calibration is assessed using calibration curves and the Hosmer–Lemeshow test. Clinical net benefit is evaluated using decision curve analysis. The fixed prediction model from the training set is then directly applied to the internal validation set (without variable selection or model reconstruction) and its discrimination, calibration and clinical utility are evaluated using ROC curves, calibration curves and decision curve analysis.

To further evaluate the model’s generalizability across different times and populations, external validation is performed. Clinical data from 300 hospitalized T2DM patients (January 1, 2021 to December 31, 2022) are collected. Using abdominal ultrasonography results as the gold standard, patients are divided into the MASLD group (n=150) and the non-MASLD group (n=150). The fixed prediction model is directly applied to this external validation set, and its discrimination, calibration and clinical net benefit are evaluated using ROC curves, calibration curves and decision curve analysis.

### Model construction and evaluation

Stratified random sampling (random seed 123) is used to divide subjects into a training set and a validation set at a 7:3 ratio. In the training set, variables are initially screened using univariate logistic regression (*P* < 0.1) and then independent predictors are determined via multivariate logistic regression (*P* < 0.05) to construct a diagnostic nomogram model. Diagnostic ROC curves are used to evaluate the independent predictors and a diagnostic nomogram is constructed based on these variables. For internal and external validation, ROC curves, calibration curves and decision curve analysis are used to comprehensively evaluate the model’s discrimination, calibration and clinical utility.

## Results

### Baseline data

A total of 470 T2DM patients are enrolled according to inclusion and exclusion criteria and divided into a Non-case group and an Case group. Comparisons between the two groups reveal statistically significant differences (*P* < 0.05) in age, weight, BMI, smoking history, fasting blood glucose, fasting insulin, C-peptide, cholesterol, triglycerides, high-density lipoprotein, low-density lipoprotein, uric acid, creatinine, urea, alanine aminotransferase, aspartate aminotransferase, gamma-glutamyltransferase, lymphocyte count, hemoglobin and PLR (**Table 1**).

**Table 1:** Baseline characteristics of patients with T2DM and NAFLD.

Characteristics	Non-case group	Case group	P value
n	240	230	
Sex, n (%)			0.856
Male	147 (31.3%)	139 (29.6%)	
Female	93 (19.8%)	91 (19.4%)	
Age, median (IQR)	62 (56, 72)	56 (48, 66)	< 0.001
Weight, median (IQR)	60.5 (54, 68)	71 (64.25, 79)	< 0.001
Height, median (IQR)	1.65 (1.58, 1.72)	1.66 (1.6, 1.73)	0.195
BMI, median (IQR)	22.309 (20.713,24.619)	25.728 (23.677,28.351)	< 0.001
Smoking, n (%)			0.023
0	213 (45.3%)	187 (39.8%)	
1	27 (5.7%)	43 (9.1%)	
Hypertensive, n (%)			0.215
0	143 (30.4%)	124 (26.4%)	
1	97 (20.6%)	106 (22.6%)	
FPG, median (IQR)	8.5 (6.4, 11.9)	9.6 (7.925, 12.3)	< 0.001
HbA1c, median (IQR)	9.5 (7.775, 11.5)	9.8 (8.4, 10.975)	0.462
Insulin, median (IQR)	3.37 (1.04, 6.3375)	7.38 (4.815, 12.45)	< 0.001
C-P, median (IQR)	1.325 (0.8275, 2.4)	2.36 (1.695, 3.2925)	< 0.001
TC, median (IQR)	4.255 (3.6425, 5.1375)	4.7 (4.2325, 5.4875)	< 0.001
TG, median (IQR)	1.145 (0.87, 1.65)	2.06 (1.49, 3.2175)	< 0.001
HDL, median (IQR)	1.075 (0.8675, 1.32)	0.92 (0.77, 1.0975)	< 0.001
LDL, median (IQR)	2.315 (1.66, 2.895)	2.765 (2.08, 3.335)	< 0.001
Urea, median (IQR)	6.95 (5.8, 9.025)	5.8 (4.8, 7)	< 0.001
Cr, median (IQR)	63.5 (52.275, 83)	57 (49, 70.5)	< 0.001
UA, median (IQR)	289 (232.75, 355.25)	328.5 (266.75, 389.75)	< 0.001
ALT, median (IQR)	18 (13, 25)	28 (18, 41.75)	< 0.001
AST, median (IQR)	19 (16, 23)	21 (18, 29)	< 0.001
GGT, median (IQR)	18 (14, 27)	30 (20, 54)	< 0.001
Alb, median (IQR)	43.1 (40.8, 45.575)	43.1 (40.35, 45.8)	0.783
Glob, median (IQR)	26.45 (24.1, 29.425)	26.2 (23.7, 29.2)	0.377
WBC, median (IQR)	6.425 (5.05, 7.88)	6.56 (5.3175, 7.67)	0.561
Neut, median (IQR)	3.83 (2.82, 4.9675)	3.835 (3.0325, 4.855)	0.877
Lymph, median (IQR)	1.775 (1.37, 2.225)	1.93 (1.54, 2.355)	0.014
Mono, median (IQR)	0.38 (0.3, 0.47)	0.39 (0.3, 0.48)	0.296
Eos, median (IQR)	0.12 (0.06, 0.19)	0.11 (0.07, 0.1775)	0.724
Baso, median (IQR)	0.03 (0.02, 0.04)	0.03 (0.02, 0.04)	0.064
Hb, median (IQR)	131 (118, 145)	140 (128.25, 154)	< 0.001
PLT, median (IQR)	187.5 (145, 231.25)	180 (148.25, 226.75)	0.459
NLR, median (IQR)	2.0509 (1.5283, 2.8698)	1.9472 (1.5702, 2.558)	0.182
PLR, median (IQR)	102.92 (81.689, 132.19)	94.995 (73.037, 120.18)	0.009
MLR, median (IQR)	0.20257 (0.15984, 0.27546)	0.20048 (0.15242, 0.24925)	0.347
SII, median (IQR)	374.45 (265.48, 561.06)	354.53 (254.56, 520.37)	0.200
SIRI, median (IQR)	0.7684 (0.50688, 1.2641)	0.7439 (0.52627, 1.1476)	0.701

In smoking and hypertension, 0 indicates no and 1 indicates yes (n=470).

Alanine aminotransferase (ALT), Aspartate aminotransferase (AST), Gamma-glutamyltransferase (GGT), Albumin (ALB), Globulin (Glob), Total cholesterol (TC), Triglycerides (TG), High-density lipoprotein cholesterol (HDL), Low-density lipoprotein cholesterol (LDL), Fasting blood glucose (FBG), Glycated hemoglobin A1c (HbA1c), C-peptide (C-P), Fasting insulin (Insulin), Urea (Urea), Creatinine (Cr), Uric acid (UA), White blood cells (WBC), Neutrophil count (Neut), Lymphocyte count (Lymph), Monocyte count (Mono), Hemoglobin (Hb), Platelets (PLT), Neutrophil-to-lymphocyte ratio (NLR), Platelet-to-lymphocyte ratio (PLR), Monocyte-to-lymphocyte ratio (MLR), Systemic immune-inflammation index (SII) and Systemic inflammatory response index (SIRI).

### Univariate and multivariate logistic regression analysis

**Figure 1:**
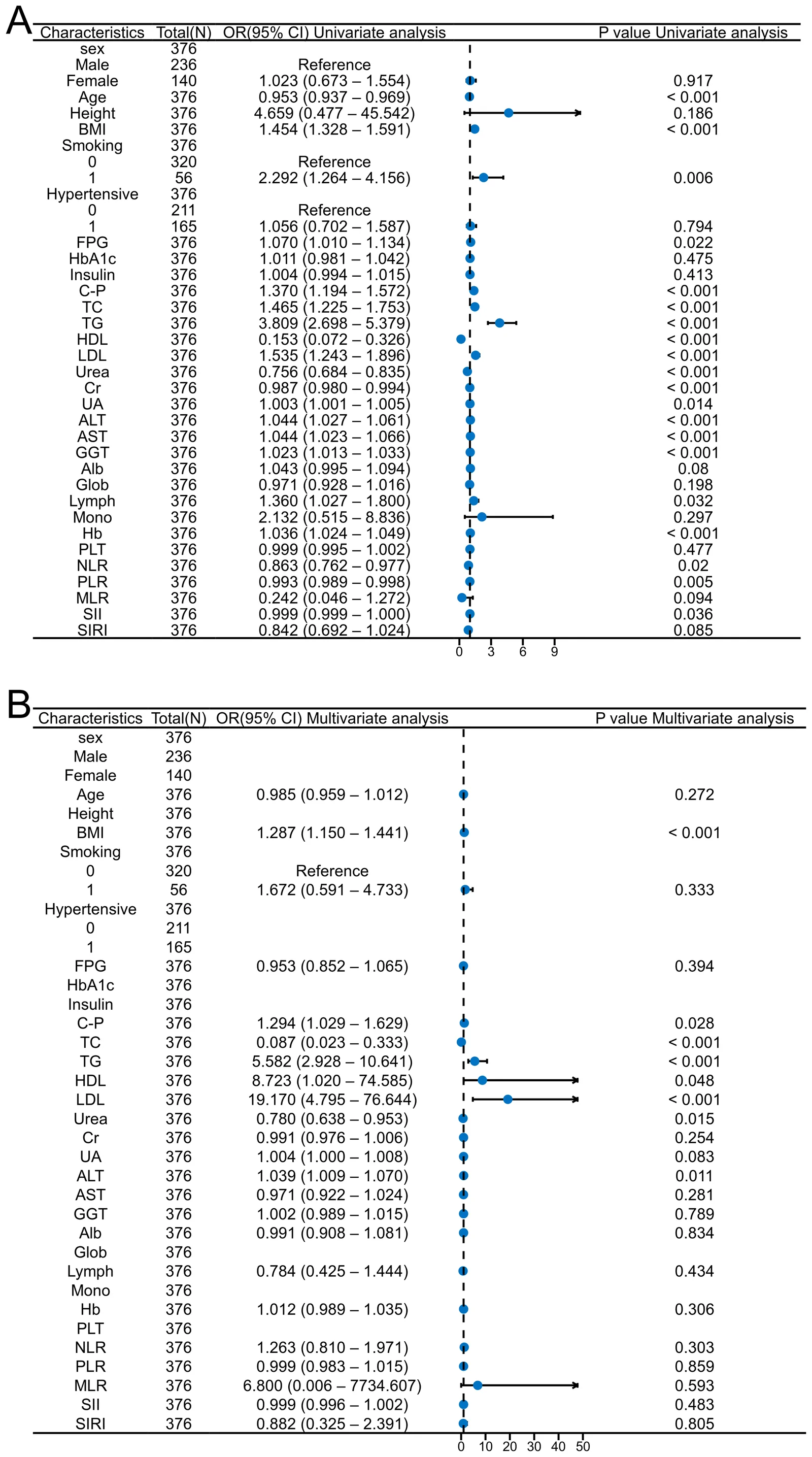
Univariate and multivariate Logistic regression analysis of T2DM patients with MASLD. **(A)** shows univariate Logistic regression analysis and **(B)** shows multivariate Logistic regression analysis. In smoking and hypertension, 0 indicates no and 1 indicates yes.

Using stratified random sampling stratified by outcome event proportion, all study samples are randomly divided into training and validation sets at a 7:3 ratio with a fixed random seed (123) to ensure reproducibility. In the training set, potential predictive factors are initially screened using univariate logistic regression. Variables with *P* < 0.1 are included in multivariate logistic regression analysis to identify statistically significant indicators and determine independent predictors. As shown in **Figure 1A**, univariate logistic regression indicated that age, BMI, smoking, fasting blood glucose, C-P, TC, TG, HDL, LDL, uric acid, creatinine, urea, alanine aminotransferase, aspartate aminotransferase, gamma-glutamyltransferase, albumin, lymphocyte count, hemoglobin, NLR, PLR and SII are statistically significant (*P* < 0.05). Variables with *P* < 0.1 are included in multivariate logistic regression. As shown in **Figure 1B**, multivariate analysis indicate that BMI, C-P, TC, TG, HDL, LDL, urea and ALT are statistically significant (*P* < 0.05) and are independent predictors of T2DM with MASLD.

### Diagnostic ROC curves

**Figure 2:**
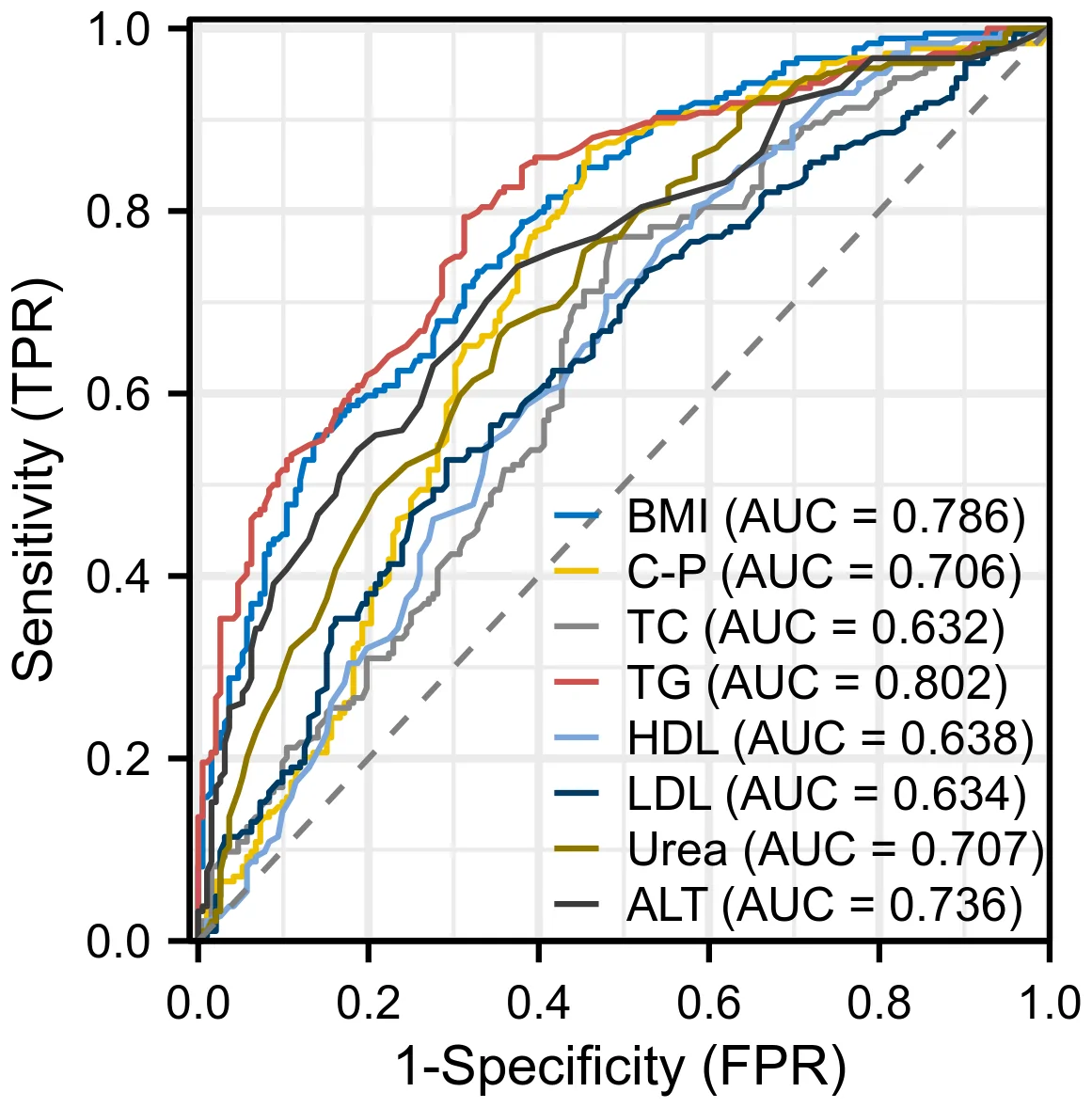
Diagnostic ROC curve of independent predictors in patients with T2DM combined with MASLD.

Following univariate and multivariate logistic regression, BMI, C-P, TC, TG, HDL, LDL, Urea and ALT are identified as independent predictors. Diagnostic ROC curves are used to evaluate their diagnostic value. As shown in **Figure 2**, the diagnostic value (assessed by AUC) from highest to lowest was: TG, BMI, ALT, urea, C-P, HDL, LDL and TC.

**Table 2** presents the optimal cut-off values and performance metrics. For BMI, the optimal cut-off is 25.49, with sensitivity of 0.55 and specificity of 0.86 (AUC 95% CI: 0.74–0.83), indicating high specificity but relatively low sensitivity. For C-P, the cut-off is 1.42, with the highest sensitivity of 0.87, specificity of 0.54 and AUC 95% CI of 0.65–0.76, suggesting strong ability to detect positive cases but lower specificity. Cut-off values for TC, TG, HDL, LDL, urea and ALT are 4.23, 1.45, 1.05, 2.76, 6.65 and 19.50, respectively, with sensitivity and specificity ranging between 0.50 and 0.80. Among these, TG has a superior AUC range (95% CI: 0.76–0.85), indicating higher overall diagnostic value. The 95% CI for each indicator do not cross 0.50, confirming the statistical significance of predictive performance.

**Table 2:** Statistical analysis results of independent predictive factors using diagnostic ROC curves (n=376).

Variables	Cut-off value	Sensitivity	Specificity	95% confidence interval (95% CI)
BMI	25.49	0.55	0.86	0.74 - 0.83
C-P	1.42	0.87	0.54	0.65 - 0.76
TC	4.23	0.77	0.51	0.58 - 0.69
TG	1.45	0.79	0.69	0.76 - 0.85
HDL	1.05	0.71	0.52	0.58 - 0.69
LDL	2.76	0.53	0.71	0.58 - 0.69
Urea	6.65	0.67	0.64	0.66 - 0.76
ALT	19.5	0.74	0.63	0.67 - 0.79

### Diagnostic nomogram

**Figure 3:**
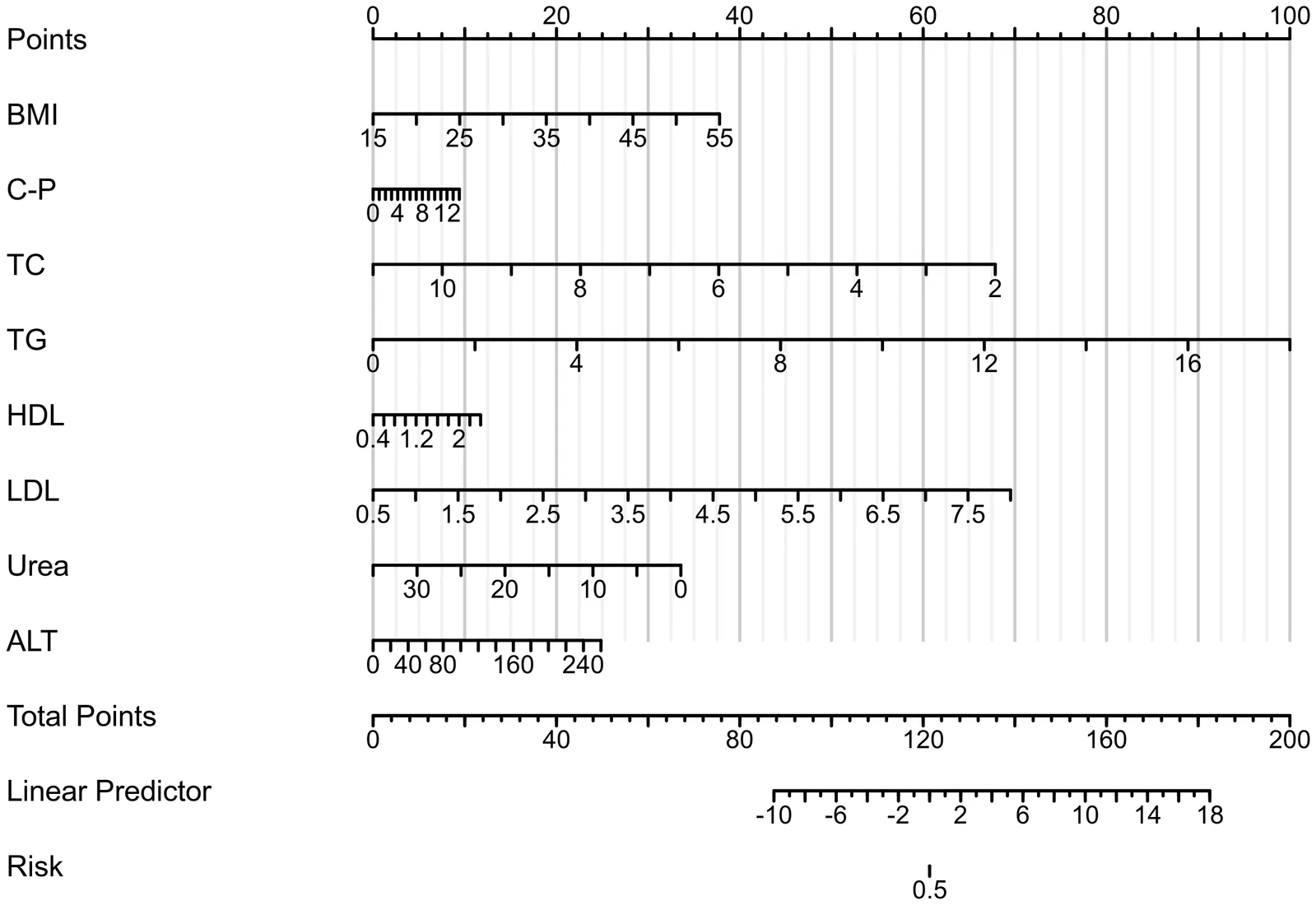
Nomogram of the diagnostic prediction model for patients with T2DM complicated by MASLD.

Based on the multivariate regression results, a diagnostic prediction model for T2DM with MASLD is constructed using the formula: Logit(P) = -7.6687 + 0.27821 × BMI + 0.19774 × C-P - 2.2205 × TC + 1.636 × TG + 1.7266 × HDL + 2.7301 × LDL - 0.28244 × urea + 0.028153 × ALT. The “rms” package in R software (version 4.2.1) is used to visualize the prediction model as a nomogram (**Figure 3**). The first row (Points) represents the risk weight scores for each independent predictor. Rows 2 to 9 correspond to the eight independent predictors: BMI, C-P, TC, TG, HDL, LDL, urea and ALT. For each laboratory measurement value, a vertical line is drawn upward to the “Points” scale to determine the individual risk score. The sum of scores for all factors (Total Points) is then mapped to the “Risk” scale in the last row, yielding the individualized risk prediction value. Higher total scores correspond to a greater predicted probability of T2DM with MASLD.

### ROC curve of the diagnostic model

**Figure 4:**
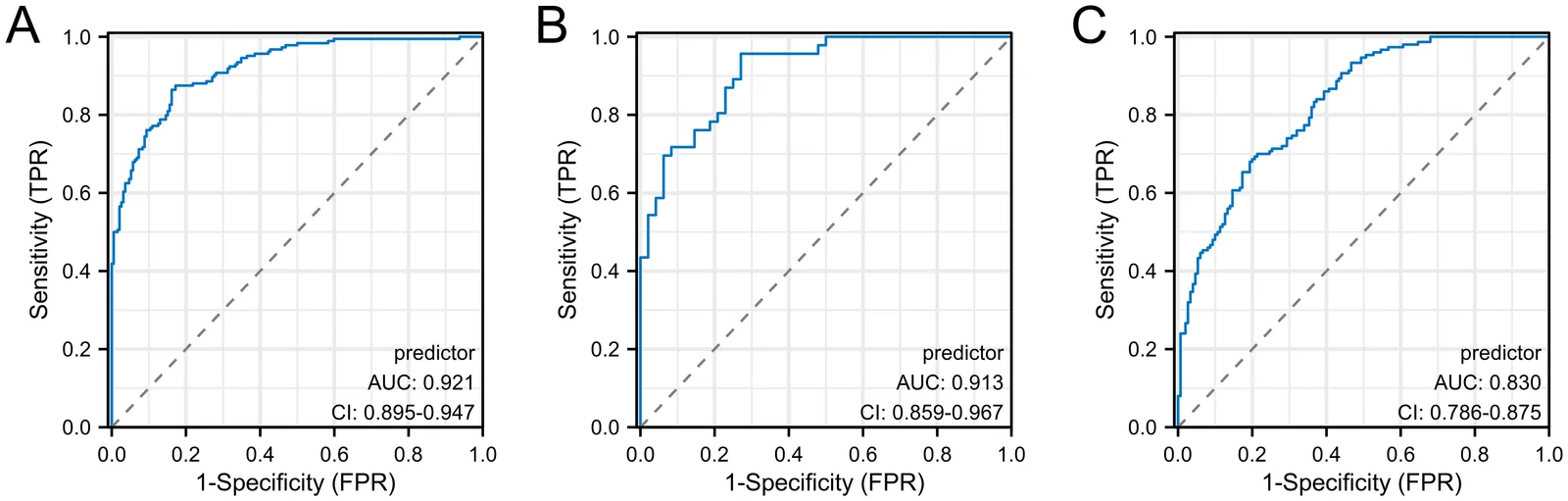
ROC curve of the diagnostic nomogram for T2DM patients with MASLD. **(A)** shows the ROC curve of the training set, **(B)** shows the ROC curve of the validation set and **(C)** shows the ROC curve of the external validation set.

To further evaluate the model’s discriminative ability, ROC curve analysis is performed. As shown in **Figure 4A**, the AUC for the training set is 0.921 (95% CI: 0.895–0.947), indicating good discriminative ability. Similarly, the validation set yielded an AUC of 0.913 (95% CI: 0.859–0.967; **Figure 4B**), demonstrating robust discriminative performance. As shown in **Figure 4C**, the AUC for the external validation set is 0.830 (95% CI: 0.786–0.875), also indicating good discriminative ability.

### Calibration curve of the diagnostic model

**Figure 5:**
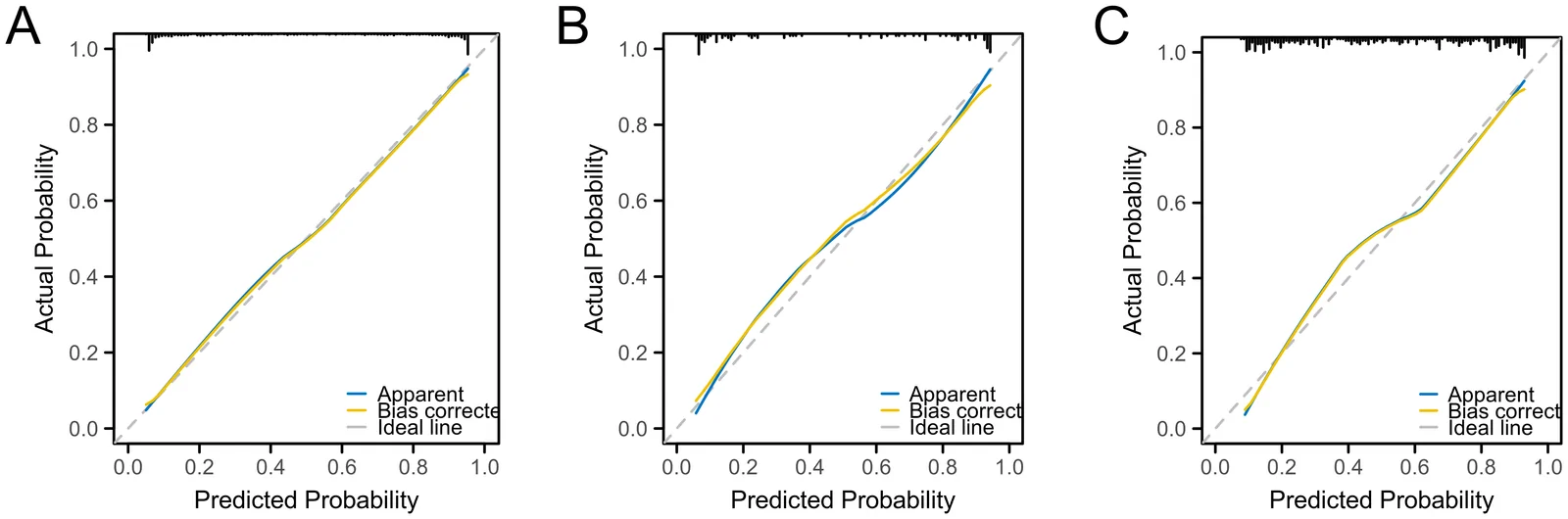
Calibration curves of the diagnostic nomogram for T2DM patients with MASLD. **(A)** shows the calibration curve of the training set, **(B)** shows the calibration curve of the validation set and **(C)** shows the calibration curve of the external validation set.

Calibration curves are used to assess model fit by depicting agreement between predicted and observed probabilities (**Figure 5**). In these plots, the “Apparent” line represents raw predictions, the “Bias-corrected” line indicates calibrated estimates and the “Ideal” line denotes perfect prediction, proximity to the diagonal indicates better fit. Calibration plots for the training (**Figure 5A**), validation (**Figure 5B**) and external validation sets (**Figure 5C**) show that bias-corrected curves closely aligned with the diagonal, suggesting high consistency between predicted and observed probabilities and indicating excellent model calibration.

### Decision curve analysis

**Figure 6:**
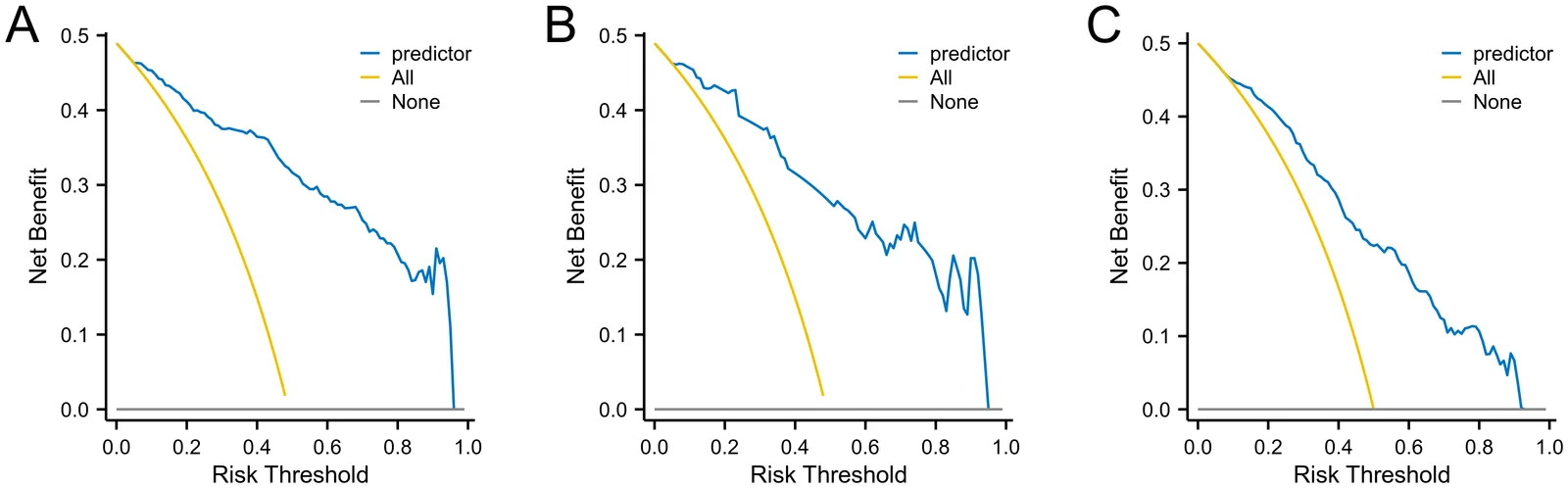
DCA plot of the diagnostic nomogram for T2DM patients with MASLD. **(A)** shows the DCA plot of the training set, **(B)** shows the DCA plot of the validation set and **(C)** shows the DCA plot of the external validation set.

Decision curve analysis (DCA) describes the change in net benefit rate when intervention is performed based on the model’s predicted value as the risk probability threshold varies (**Figure 6**). The model demonstrates a high net benefit across threshold probabilities ranging from 0.01 to 0.95 in the training set (**Figure 6A**), 0.01 to 0.94 in the validation set (**Figure 6B**) and 0.07 to 0.92 in the external validation set (**Figure 6C**).

## Discussion

There is a close bidirectional pathophysiological relationship between MASLD and T2DM. They often act as cause and effect for each other, jointly driven by insulin resistance and lipid metabolism disorders (12). MASLD is not only considered a hepatic manifestation of metabolic syndrome but also an important independent risk factor for T2DM development (13). Although liver biopsy is the gold standard for diagnosis, its invasiveness and sampling variability limit routine screening; existing imaging modalities, while widely used, lack quantitative assessment and are operator-dependent (14). Therefore, developing efficient, non-invasive diagnostic tools based on routine clinical indicators is of urgent clinical significance for early identification and intervention in this comorbid population.

Addressing this challenge, this study constructs and validates a diagnostic model relying solely on routine serological indicators and anthropometric parameters, aiming to provide an economical, accessible and highly accurate screening strategy for T2DM complicated by MASLD. The results show that BMI, C-P, lipids, urea and ALT are independent predictors of this comorbidity. The derived nomogram model demonstrates excellent discrimination (AUC > 0.91) and calibration in both the training and validation sets. Decision curve analysis further confirms that the model offers significant clinical net benefit across a wide range of threshold probabilities, outperforming traditional single indicators or complex scoring systems.

In the metabolic risk profile of T2DM complicated by MASLD, elevated triglyceride (TG) levels are identified as one of the most significant independent predictors, ranking highest in importance in random forest analysis. From a pathophysiological perspective, excessive accumulation of circulating TG reflects a severe imbalance in hepatic lipid metabolism. In the setting of insulin resistance, enhanced lipolysis in peripheral adipose tissue releases a substantial influx of free fatty acids (FFAs) into the liver. This influx exceeds the compensatory capacity of hepatocyte mitochondrial β-oxidation, resulting in significant ectopic triglyceride deposition within hepatocytes—a core initiating step in MASLD pathogenesis (15). Concurrently, hyperinsulinemia persistently activates sterol regulatory element-binding protein-1c (SREBP-1c), promoting hepatic *de novo* lipogenesis and further exacerbating intrahepatic TG synthesis and accumulation (16). Incorporating this routine lipid indicator as a core variable in the model aligns with findings from a study on T2DM patients, which identify elevated TG as an independent predictor of metabolic-associated fatty liver disease (17). Another retrospective study confirms that high TG is an independent risk factor for MASLD in T2DM patients (18). By employing the random forest algorithm, this study quantifies the superior importance of TG relative to other indicators, elucidating its dominant role in the development of this comorbidity. In summary, as a core marker reflecting hepatic lipotoxicity, the high weight of TG in this model has a solid pathophysiological basis.

In this study, urea, a surrogate marker of renal function and protein metabolism, emerge as a protective factor for T2DM complicated by MASLD in the statistical model. This finding reveals a noteworthy metabolic intersection. From a pathophysiological perspective, decreased urea levels may not simply reflect improved renal function but rather indirectly indicate metabolic remodeling of the hepatic urea cycle. Under MASLD conditions, the hepatic urea cycle may undergo adaptive changes due to mitochondrial dysfunction and metabolic stress. Additionally, studies suggest that gut microbiota urease activity may be altered in MASLD patients, affecting ammonia recycling in the enterohepatic circulation and reducing the substrate load for hepatic urea production. At a deeper mechanistic level, this relative decrease in urea levels may relate to enhanced systemic protein catabolism and redistribution of gluconeogenic substrates in T2DM with MASLD. A large cross-sectional study exploring the mechanisms of T2DM and MASLD comorbidity finds that the presence of MASLD significantly altered the overall metabolomic profile of patients, including multiple markers related to amino acid metabolic pathways (19). Another study on the relationship between MASLD fibrosis and chronic kidney disease in T2DM shows a positive additive interaction between increased risk of liver fibrosis and decreased renal function (20). These findings collectively suggest a complex metabolic coupling between the liver and kidneys in the metabolic disorder of T2DM with MASLD. Although urea shows a negative correlation in the model, the driving mechanism may not be a simple protective effect but rather reflects a combined result of compensatory and decompensatory changes in the liver-kidney metabolic axis under disease conditions.

Among liver function-related indicators, alanine aminotransferase (ALT), an independent predictor in this model, directly reflects the degree of hepatocyte injury. Elevated ALT is not merely enzymatic leakage; in the pathological context of MASLD, it represents lipotoxic hepatocyte damage caused by the accumulation of fatty acid metabolic intermediates (such as diacylglycerol and ceramides) (21). These lipid intermediates can activate protein kinase C (PKC) isoforms, subsequently inhibiting phosphorylation of insulin receptor substrates and exacerbating local and systemic insulin resistance. This study finds that ALT levels in T2DM patients with MASLD are significantly higher than in those with T2DM alone, consistent with previous reports, confirming ALT as a sensitive indicator for distinguishing the two groups (22). However, reliance solely on ALT has limitations, as some patients with biopsy-confirmed NASH may have normal ALT levels (23). By combining ALT with metabolic indicators such as TG and BMI, this model overcomes the insufficient sensitivity of a single liver enzyme indicator while simultaneously capturing liver injury and metabolic burden at the mechanistic level, thereby enhancing overall diagnostic accuracy.

This study identifies BMI as a strong predictor of MASLD, with its core mechanism lying in adipose tissue dysfunction and ectopic fat deposition under obesity. Chronic energy intake excess, leading to saturation of subcutaneous fat storage capacity, causes adipocyte hypertrophy, accompanied by substantial secretion of pro-inflammatory factors (such as tumor necrosis factor-α, interleukin-6) and a significant decrease in adiponectin levels (24). This imbalance in adipokines directly leads to insulin resistance in the liver and skeletal muscle and promotes ectopic deposition of FFAs into non-adipose tissues such as the liver and pancreas. This study shows that BMI is significantly higher in the MASLD group than in the Non-case group, providing clinical data support for the above mechanism. This finding is consistent with a meta-analysis covering a large sample of T2DM patients, which identifies obesity as one of the main driving factors for MASLD in T2DM patients (25). Furthermore, a study on a Ghanaian population emphasizes that obesity is an independent predictor of MASLD in T2DM patients, consistent with our findings (26). By incorporating BMI, a simple anthropometric indicator, the diagnostic model constructed in this study provides clinicians with an intuitive risk stratification tool.

In the pathological state of MASLD, there is a bidirectional regulatory relationship between inflammation and the urea cycle. On one hand, pro-inflammatory factors, by activating the NF-κB/JNK pathway, directly downregulate the expression of key urea cycle enzymes CPS1 and OTC (27), impairing hepatic ammonia clearance and leading to ammonia accumulation (28). On the other hand, high concentrations of ammonia can induce NLRP3 inflammasome activation in hepatic stellate cells and Kupffer cells, promoting IL-1βsecretion and amplifying the inflammatory response (29); furthermore, metabolic imbalance of the urea cycle intermediate arginine modulates inflammatory signaling pathways such as NF-κB and mTOR (30). This positive feedback loop of “inflammation → urea cycle inhibition → ammonia accumulation → exacerbated inflammation” is a key pathological mechanism driving the progression of MASLD from simple steatosis to NASH and fibrosis.

This study uses abdominal ultrasound as the diagnostic basis for MASLD. Although ultrasound is a widely used screening tool in frontline clinical practice, its sensitivity for mild hepatic steatosis is limited and it cannot distinguish simple steatosis from NASH or hepatic fibrosis (31). This may lead to the misclassification of cases involving patients with mild steatosis, resulting in outcome misclassification. Such non-differential misclassification typically leads to an underestimation of the true strength of association between predictive factors and the disease. Consequently, the effect sizes of certain predictors may be attenuated, or the model’s overall discriminatory power (AUC) may fall below its true potential. Therefore, future studies should employ more accurate quantitative methods, such as MRI-PDFF or controlled attenuation parameters derived from transient elastography, to minimize measurement bias and further evaluate the model’s capacity to distinguish NASH from simple steatosis.

This study does not collect detailed data on patients’ use of antidiabetic drugs (particularly GLP-1 receptor agonists, SGLT2 inhibitors, metformin and pioglitazone), lipid-lowering medications (such as statins) or vitamin E. These drugs are known to significantly influence liver fat content, lipid profiles, ALT and fasting C-peptide levels, potentially introducing bias into the model’s predictive performance (32). Therefore, these unmeasured confounding factors constitute a major limitation of this study. Future research should prospectively collect medication history and include them as covariates in the model to control for these potential confounding effects, to enable a more accurate assessment of the independent roles of predictive factors.

Given the close relationship between T2DM, MASLD and cardiovascular diseases, the clinical significance of this model lies not only in screening for liver disease but also in its indication of overall cardiovascular and metabolic risk. Recent studies have emphasized the core role of cardiovascular prevention, inflammation and lifestyle interventions in the management of metabolic diseases (33, 34), which aligns with the concept of this model—integrating multiple metabolic indicators. Notably, these studies collectively highlight that chronic low-grade inflammation is the shared pathophysiological basis connecting metabolic disorders, cardiovascular events and liver disease. The indicators included in this model (such as BMI, TG and HDL) themselves reflect systemic inflammatory status and insulin resistance. Our model can serve as a screening tool to accurately stratify high-risk patients most likely to benefit from intensive lifestyle interventions.

In addition, the diagnostic model constructed in this study has other limitations. First, the study adopts a retrospective design, which inevitably introduce selection bias. Second, the diagnosis of MASLD is based on abdominal ultrasonography rather than the gold standard liver biopsy, which may underestimate or overestimate the severity of hepatic steatosis, especially in patients with mild steatosis. Third, this is a single-center study with limited data sources, which restricts the external generalizability of the model. Finally, this study does not compare the proposed model with existing non-invasive scores such as the Fatty Liver Index (FLI), Hepatic Steatosis Index (HSI) and other established MASLD prediction models. Therefore, future prospective validation in multicenter, multi-ethnic and geographically diverse populations is warranted to assess the model’s generalizability and temporal stability. Furthermore, future comparisons with existing non-invasive scores such as the Fatty Liver Index (FLI), Hepatic Steatosis Index (HSI) and other established MASLD prediction models are also necessary. To enhance discriminatory ability, future research could integrate novel biomarkers. For example, cytokeratin-18 fragment (CK-18 M30), a marker of hepatocyte apoptosis, has been associated with MASLD and metabolic diseases. Fibroblast growth factor-21 (FGF-21) plays an important role in glucose and lipid metabolism and its level changes are closely related to MASLD development. Additionally, epigenetic markers such as microRNA-122 (miR-122) are considered potential MASLD risk markers. Combining these novel biomarkers with routine serological indicators is expected to yield diagnostic models with better predictive performance and higher clinical application value.

In summary, this study successfully constructs and internally validates a high-precision nomogram model (AUC > 0.9) for diagnosing T2DM complicated by MASLD, based on BMI and seven routine serological indicators. Its non-invasive, convenient and low-cost features align closely with the urgent clinical need for efficient screening tools, providing a practical approach for early stratification of liver comorbidity risk in T2DM patients. This achievement may reduce unnecessary invasive examinations and optimize medical resource allocation. Future efforts should focus on external validation in multicenter, prospective cohorts and explore the model’s association with liver histological severity and long-term liver and cardiovascular outcomes, ultimately promoting its use as a standardized screening tool in routine T2DM management and improving overall patient prognosis.

## Conclusion

This study is based on a retrospective case-control design, enrolling 470 patients with T2DM (the training set and internal validation set are stratified by 7:3 random sampling) and an additional 300 patients are collected for external validation. The aim is to construct and validate a simple, non-invasive diagnostic model for T2DM complicated by MASLD using routine serological indicators. Through univariate and multivariate Logistic regression analysis, eight independent predictive factors are identified: BMI, C-P, TC, TG, HDL, LDL, Urea and ALT. These are used to construct a nomogram. The AUC of the model in the training set, internal validation set and external validation set is 0.921, 0.913 and 0.830, respectively. The calibration curve is close to the ideal diagonal line, and decision curve analysis shows significant clinical net benefit across a wide threshold range, indicating good discrimination, calibration and clinical utility of the model. The study confirms that high-precision non-invasive diagnosis of T2DM complicated by MASLD can be achieved using routine and easily accessible serological indicators, overcoming the limitations of invasive liver biopsy and providing an economical, convenient and reliable tool for frontline clinical screening. The successful construction of this model addresses the scarcity of simple diagnostic tools in the context of T2DM comorbidity and has significant potential for clinical translation. Future studies need to further validate its generalizability in multicenter, prospective cohorts and explore its association with liver histological severity and long-term prognosis, to promote its integration as a standardized screening strategy in routine management of T2DM patients, ultimately improving overall patient outcomes.

## Data Availability

The raw data supporting the conclusions of this article will be made available by the authors, without undue reservation.
